# Case report: HLA-haploidentical hematopoietic cell transplant with posttransplant cyclophosphamide in a patient with leukocyte adhesion deficiency type I

**DOI:** 10.3389/fimmu.2022.1020362

**Published:** 2022-10-24

**Authors:** Motoi Yamashita, Shiori Eguchi, Dan Tomomasa, Takahiro Kamiya, Daiki Niizato, Noriko Mitsuiki, Takeshi Isoda, Hanako Funakoshi, Yuki Mizuno, Kentaro Okamoto, Tuan Minh Nguyen, Hidetoshi Takada, Masatoshi Takagi, Kohsuke Imai, Tomohiro Morio, Hirokazu Kanegane

**Affiliations:** ^1^ Department of Pediatrics and Developmental Biology, Graduate School of Medical and Dental Sciences, Tokyo Medical and Dental University (TMDU), Tokyo, Japan; ^2^ Division of Clinical Management, Clinical Research Center, Tokyo Medical and Dental University (TMDU), Tokyo, Japan; ^3^ Division of Infectious Diseases, Department of Pediatrics, Tokyo Metropolitan Children’s Medical Center, Tokyo, Japan; ^4^ Department of Pediatric Surgery, Tokyo Medical and Dental University Hospital, Tokyo, Japan; ^5^ Department of Hematology, Children’s Hospital 1, HCM, Vietnam; ^6^ Department of Child Health, Faculty of Medicine, University of Tsukuba, Tsukuba, Japan; ^7^ Department of Community Pediatrics, Perinatal and Maternal Medicine, Tokyo Medical and Dental University (TUDU), Tokyo, Japan; ^8^ Department of Pediatrics, National Defense Medical College, Tokorozawa, Japan; ^9^ Department of Child Health and Development, Graduate School of Medical and Dental Sciences, Tokyo Medical and Dental University (TMDU), Tokyo, Japan

**Keywords:** leukocyte adhesion deficiency, posttransplant cyclophosphamide, hematopoietic cell transplantation, graft-versus-host disease, inborn errors of immunity

## Abstract

Leukocyte adhesion deficiency type I (LAD-I) is a rare autosomal recessive inborn error of immunity (IEI) caused by the defects in CD18, encoded by the *ITGB2* gene. LAD-I is characterized by defective leukocyte adhesion to the vascular endothelium and impaired migration of leukocytes. Allogeneic hematopoietic cell transplant (HCT) is the only curative treatment for LAD-I. In an absence of ideal donor for HCT, human leukocyte antigen (HLA)-haploidentical HCT is performed. Posttransplant cyclophosphamide (PT-CY) is a relatively new graft-versus-host disease (GVHD) prophylactic measure and has been increasingly used in HLA-haploidentical HCT for malignant and nonmalignant diseases. However, experience in using PT-CY for rare IEIs, such as LAD-I, is very limited. We report a case of LAD-I successfully treated with HLA-haploidentical HCT with PT-CY. Complete chimerism was achieved, and the patient was cured. Her transplant course was complicated by mild GVHD, cytomegalovirus reactivation and veno-occlusive disease/sinusoidal obstruction syndrome, which were successfully treated. HLA-haploidentical HCT with PT-CY is a safe and effective option for patients with LAD-I when HLA-matched donors are unavailable.

## Introduction

Neutrophils play a central role in the innate immune response against pathogens like bacteria and fungi. Extravasation of circulating neutrophils is the crucial step in the first response to various chemokines secreted from the infection site. This trafficking is mediated by adhesive interactions between neutrophils and the vascular endothelium. Neutrophils and vascular endothelial cells express adhesive molecules, defects in which cause leukocyte adhesion deficiencies (LADs). LAD-I results from biallelic loss-of-function (LOF) variants in the *ITGB2* gene, which encodes the CD18 molecule. CD18 forms β2 integrins with its heterodimerizing partners CD11b and CD11c on the neutrophil surface. Upon activation, β2 integrins bind to their ligands expressed on the vascular endothelium (i.e., intracellular adhesion molecules). The phenotypic severity of LAD-I correlates with the degree of CD18 deficiency. Severe LAD-I is defined as <2% of neutrophils expressing CD18 as assessed by flow cytometric analysis. Patients with severe LAD-I suffer from recurrent life-threatening bacterial and fungal infections, often leading to death in infancy unless curative treatment is performed (1). LAD-II and LAD-III are rare LADs that result from biallelic LOF variants in the *SLC35C1* and *FERMT3* genes, respectively. The *SLC35C1* gene encodes a fucose transporter to the Golgi apparatus, and the defects in this transporter result in the defective fucosylation of glycoproteins (CD15a), which are ligands for selectins (2). The *FERMT3* gene encodes Kindlin-3, which plays a major role in regulating the activation of all β-integrins (3, 4).

The current curative treatment for LAD-I and LAD-III is hematopoietic cell transplant (HCT). A human leukocyte antigen (HLA)-identical sibling is considered the ideal donor for HCT. In the absence of HLA-identical siblings, HLA-matched and unmatched unrelated donors or umbilical cord blood (UCB) are the alternative options. However, the lack of available donors has been a frequent problem. Thus, the utilization of HLA-haploidentical donors became an attractive option. The high rate of graft-versus-host disease (GVHD) has been the major obstacle in using haploidentical donors for HCT. Administration of high-dose posttransplant cyclophosphamide (PT-CY) strongly suppresses acute and chronic GVHD. PT-CY depletes alloreactive T cells in the recipient, whereas regulatory T cells and hematopoietic stem cells are spared (5, 6). Alternatively, *ex vivo* T-cell depletion techniques, such as T-cell receptor-α/β^+^ and CD19^+^ cell depletion and CD34^+^ cell selection of the grafts, have been used, but these manipulations are highly expensive and require specific equipment. PT-CY has been increasingly used as the strong GVHD prophylactic measures, especially in haploidentical HCT. Although an increasing number of HCT with PT-CY are performed in malignant and nonmalignant diseases, experiences in rare inborn errors of immunity (IEIs), such as LAD, are still limited (7–11). We present a case of a LAD-I patient successfully cured by haploidentical HCT with PT-CY.

## Results

### Case report

**Figure 1:**
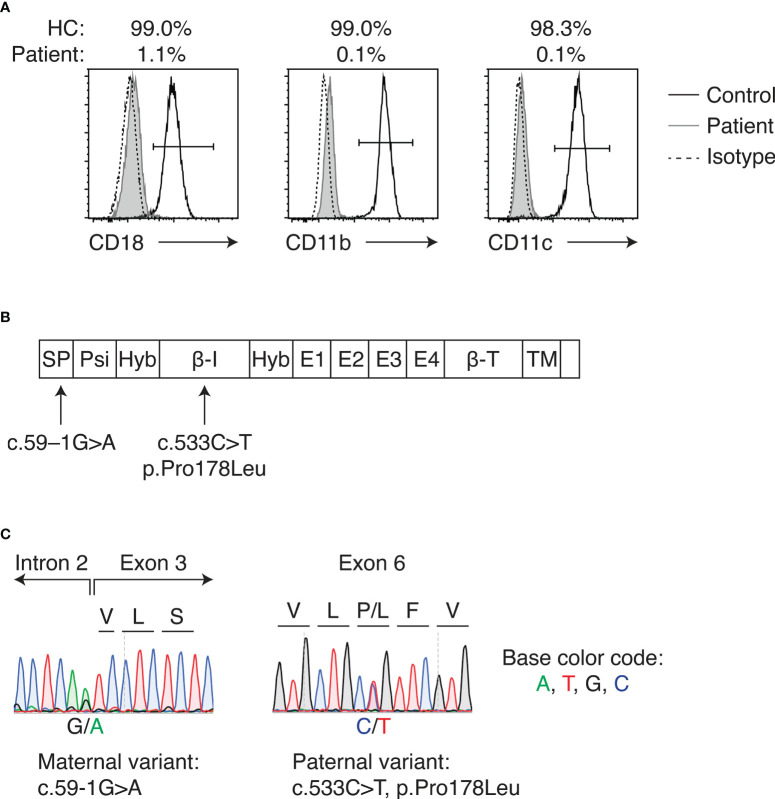
LAD-I diagnosis in the patient. **(A)** Flow cytometric analysis of CD18, CD11b, and CD11c expression on peripheral blood neutrophils of the patient and healthy control. Percentages above the plots indicate the percent positive cells for each molecule, as gated by the bars in each plot. **(B)** Domains of CD18 and locations of the variants detected in the patient. **(C)** Sanger sequencing of identified *ITGB2* variants. Letters above the plot indicate amino acids. HC, healthy control; SP, signal peptide; Psi, plexin-semaphorin-integrin; Hyb, hybrid; β-I, I-like; E, integrin epidermal growth factor; β-T, β-tail; TM, transmembrane.

A 5-month-old Vietnamese girl was referred to our hospital for the evaluation of recurrent omphalitis (**Supplementary Figure 1**). She was born to nonconsanguineous Vietnamese parents, and no family history of immunodeficiency was noted. The separation of the umbilical cord occurred at 14 days of life. She developed omphalitis at 6 days, 1 month and 4 months of life, each of which was treated with intravenous (IV) antibiotics. Her past medical history included the urachal remnant and grade III vesicoureteral reflux. At presentation, she was febrile without an obvious focus of infection. The laboratory study revealed marked leukocytosis (white blood cells 90,970/μL, neutrophils 67,770/μL, and lymphocytes 18,190/μL) and elevated C-reactive protein level (15.8 mg/dL). As the history of recurrent omphalitis and the significant leukocytosis suggested LAD as the differential diagnosis, surface expression of CD11b, CD11c, and CD18 on peripheral blood neutrophils was examined by flow cytometry. CD18 expression was significantly decreased, showing 1.1% of neutrophils were positive for CD18 expression (**Figure 1A**). CD11b and CD11c expression on the neutrophils also decreased. Based on her clinical phenotype and flow cytometric analysis, LAD-I was suspected and the target gene panel of LAD (12) was performed. Two variants, namely c.533C>T, p.Pro178Leu and c.59-1G>A (NM_000211.5, NP_000202.3) in the *ITGB2* gene, were identified (**Figure 1B**). Parenteral sequencing confirmed that the variants were compound heterozygous: c.533C>T and c.59-1G>A were inherited from her father and mother, respectively (**Figure 1C**). The paternal c.533C>T variant was reported to be pathogenic (13). The maternal c.59-1G>A was unreported in LAD-I, but the variant was considered to cause splicing abnormalities since it was located at the exon-intron junction. The patient was diagnosed with severe LAD-I based on genetic studies and flow cytometric analysis demonstrating loss of CD18 expression on the neutrophils. Prophylactic antimicrobial therapy with trimethoprim/sulfamethoxazole was initiated. The patient underwent surgical excision of the urachal remnant when she was 10 months old. However, she repeated bacterial infections with an unknown focus.

**Figure 2:**
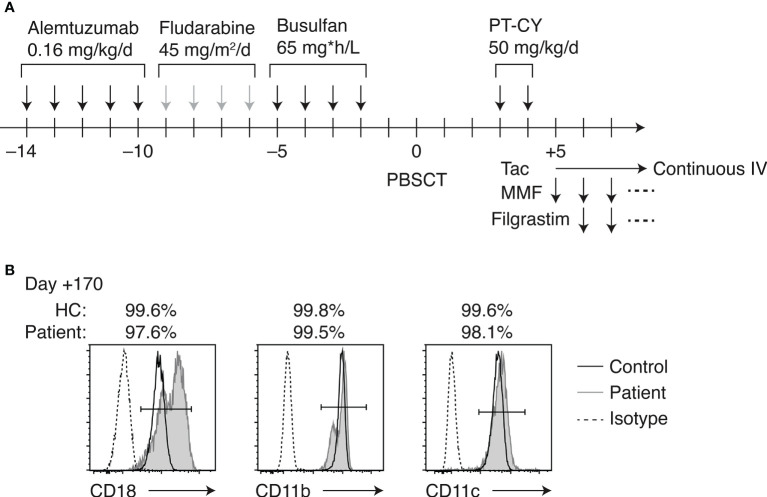
HLA-haploidentical HCT. **(A)** Conditioning regimen and GVHD prophylaxis. **(B)** Flow cytometric analysis of CD18, CD11b, and CD11c expression on peripheral blood neutrophils of the patient on day +170 of HCT.

HCT was indicated given her clinical course and severe LAD-I diagnosis. However, the patient did not have siblings. Also, there were no HLA-matched UCB or unrelated bone marrow/peripheral blood stem cell (PBSC) donors in the Japan Marrow Donor Program registry. Her father was a hepatitis B virus carrier. Thus, the HLA-haploidentical mother was chosen as the donor for HCT. Anti-HLA antibodies in the patient were tested negative. Reduced-intensity conditioning (RIC) consisted of alemtuzumab 0.8 mg/kg IV (0.16 mg/kg for 5 days; days –14 to –10), fludarabine 180 mg/m^2^ IV (45 mg/m^2^ for 4 days; days –9 to –6), and area under the curve (AUC)-targeted busulfan IV (65 mg*h/L; days –5 to –2) (**Figure 2A**). PBSCs were freshly harvested from her mother. GVHD prophylaxis included PT-CY 50 mg/kg IV on days +3 and +4, followed by tacrolimus continuous IV and oral mycophenolate mofetil (MMF) started on day +5. Filgrastim 300 μg/m^2^ IV was administered on days +6 to +12. Engraftment was achieved on day +13 of transplant. The patient experienced grade I acute GVHD (skin stage 1, liver stage 0, and gut stage 0). Her skin GVHD was successfully treated with a topical steroid. MMF was discontinued on day +42 of HCT because her GVHD was well controlled. Complete donor chimerism was observed at 1, 2 and 5 months post-HCT. The patient has been well without complications except mild occasional skin GVHD, which has been treated with topical steroids.

Cytomegalovirus (CMV) viremia was noted on day –1 of HCT, when the weekly polymerase chain reaction (PCR)-based screening of viremia was started. Ganciclovir was started on day +2 of HCT. No evident CMV disease occurred, and CMV viremia was resolved on day +54. Also, *Aspergillus* antigenemia was detected on day +54 of HCT after an increase in β-D-glucan levels. The focus of infection was not identified, but she was treated with voriconazole. In addition to those infectious complications, the patient developed veno-occlusive disease/sinusoidal obstruction syndrome (VOD/SOS) on day +24 of HCT. After a slight increase in the total bilirubin level (0.3 mg/dL at a basal level to 0.7 mg/dL), weight gain (8% increase from the basal level) over 3 consecutive days, and transfusion-refractory thrombocytopenia, gallbladder wall thickening and slight ascites were noted by ultrasonography, fulfilling the diagnostic criteria of VOD/SOS (14). The patient remained asymptomatic. Defibrotide was started on day +24 of HCT, which resolved the weight gain, increased bilirubin levels, and ultrasonographic findings. Defibrotide was discontinued on day +56 of HCT.

### Immune reconstruction after HCT

The patient’s CD18-expressing neutrophils recovered to >99% on day +28 of HCT, and simultaneous whole-blood chimerism was assessed by short tandem repeat PCR, indicating complete donor chimerism. The percentages of CD18-, CD11b-, and CD11c-expressing neutrophils remained >99% over 5 months post-HCT (**Figure 2B**). The peripheral blood lymphocyte subsets were studied throughout HCT in this patient (**Supplementary Figure 2A**). At 1 month post-HCT, B cell number was not recovered, while natural killer (NK) cell expanded. B cells became detectable in the peripheral blood at 2 months post-HCT, accompanied by the detection of κ-deleting excision circles (KRECs) in the whole blood (**Supplementary Figure 2B**). CD45RA^+^ naive T cell fraction was transiently decreased post-HCT, which gradually recovered over 5 months. T-cell receptor excision circles (TRECs) remained undetectable at 5 months, which was normalized at 10 months post-HCT. The patient required immunoglobulin supplementation until 3 months post-HCT. Oral immunosuppressive therapy with tacrolimus was continued until day +215 of HCT as the patient developed occasional dermatitis, which was considered skin GVHD.

## Discussion

LAD-I is a rare IEI characterized by defects in the migration of neutrophils. The phenotypic severity varies, and severe LAD-I (defined as CD18-expressing neutrophils <2%) has substantial infant mortality and requires prompt curative treatment (1, 15). Currently, HCT serves as the sole curative treatment option, whereas a phase 1/2 clinical trial of lentiviral-mediated gene therapy for LAD-I is undergoing (16). Patients who received HCT generally showed >80% long-term overall survival (OS) (17–20). Especially, a recent study showed >90% of 3-year OS among patients who were transplanted from HLA-identical sibling or HLA-matched unrelated donors in infancy (19). However, improvements need to be made in HLA-haploidentical HCT for patients with LAD. A study of all published LAD-I cases between 1975 and 2017 revealed that 22 patients received HLA-haploidentical HCT (15). In this cohort of patients who received haploidentical HCT, 32% of patients had HCT-related mortality, and 55% of patients received subsequent HCT. To be noted, majority of these cases utilized *ex vivo* T-cell-depleted grafts. More recently, HLA-haploidentical HCT with PT-CY began to be performed in LAD-I patients. To date, five LAD-I cases were reported to receive HLA-haploidentical HCT with PT-CY (7–9, 21). Among these reports, Neven et al. and Khandelwal et al. described the details of HCT and outcomes (9, 21). The summary of these two cases and our case is shown in **Table 1**. All three cases were cured of LAD-I, achieving 100% donor chimerism. No GVHD was observed in the previously reported two cases, whereas the present case developed mild skin GVHD. All patients experienced CMV reactivation. These data suggest that the strong immunosuppressive effect of PT-CY possibly plays a role in CMV reactivation. Regarding PT-CY-related toxicities, none but the present case developed VOD/SOS, which was successfully treated with defibrotide. Although AUC was targeted, the use of busulfan in the conditioning may have played a role in developing VOD/SOS in the present case. To reduce the risk of VOD/SOS, the use of treosulfan for the conditioning would be an alternative option for the conditioning with PT-CY. Although data are limited and more cases need to be accumulated, these observations indicate that PT-CY is an effective GVHD prophylactic measure in HLA-haploidentical HCT for LAD-I.

**Table 1:** Summary of reported HLA-haploidentical HCT with PT-CY for LAD-I patients.

	Khandewal et al. (21)	Neven et al. (9)	Patient
Severity	Severe	ND	Severe
Infectious complications pre-HCT	Sepsis	CMV infection	No
Age at HCT	15 months	3 months	12 months
Donor	32-year-old father	Father	21-year-old mother
Donor source	PBSC	BM	PBSC
CD34^+^ cells	10 × 10^6^/kg	ND	10 × 10^6^/kg
CD3^+^ cells	2.4 × 10^8^/kg	ND	2.0 × 10^8^/kg
Conditioning	RIC, Flu 160 mg/m^2^, Treo 42 mg/m^2^	MAC, Flu 160 mg/m^2^, Bu AUC adjusted to 65~100 mg*h/L	RIC, Flu 180 mg/m^2^, Bu AUC adjusted to 65 mg*h/L
Serotherapy	No	Alem 0.5 mg/kg (days –11 and –10), RTX 375 mg/m^2^ on day –12	Alem 0.8 mg/kg (days –14 to –10)
GVHD prophylaxis			
PT-CY	50 mg/kg/day on days +3 and +4	50 mg/kg/day on days +3 and +4	50 mg/kg/day on days +3 and +4
Immunosuppressants	CyA, MMF	CyA, MMF	Tac, MMF
Outcome	Cured	Cured	Cured
Engraftment	Day +15	Day +24	Day +13
Chimerism	100% donor chimerism	100% donor chimerism	100% donor chimerism
GVHD	No	No	Acute Grade I (skin stage 1, and liver and gut stage 0)
Infectious complications	CMV reactivation	CMV reactivation	CMV reactivation, aspergillosis
Other complications	No	AIHA	VOD/SOS

AIHA, autoimmune hemolytic anemia; Alem, alemtuzumab; AUC, area under the curve; BM, bone marrow; Bu, busulfan; CMV, cytomegalovirus; CyA, cyclosporine; Flu, fludarabine; GVHD, graft-versus-host disease; HCT, hematopoietic cell transplantation; MAC, myeloablative conditioning; MMF, mycophenolate mofetil; ND, not determined; PBSC, peripheral blood stem cells; PT-CY, posttransplant cyclophosphamide; RIC, reduced-intensity conditioning; RTX, rituximab; Treo, treosulfan; VOD/SOS, veno-occlusive disease/sinusoidal obstruction syndrome.

The optimal conditioning regimen for LAD-I is yet to be determined. Early studies favored myeloablative reduced-toxicity regimens in fit patients without significant comorbidities, whereas RIC was preferred in patients with worse performance status. However, the recent European Society for Blood and Marrow Transplantation (EBMT) report did not favor the myeloablative regimen (19). The current EBMT/European Society for Immunodeficiencies recommendation of the conditioning for LAD is RIC (160-180 mg/m^2^ fludarabine + busulfan with AUC of 60-70 mg*h/L, or 150-160 mg/m^2^ fludarabine + 30-42 mg/m^2^ treosulfan) (22). Relatively high frequencies of graft rejection (17%-18%) and grades II to IV acute GVHD (24%-25%), which are thought to result from the inflammatory condition in LAD patients, have been observed (15, 19). Thus, the degree of immunosuppression should be carefully determined in HCT for LAD. It has been suggested that the use of thiotepa in the conditioning might be beneficial for its anti-inflammatory aspect (23). In our case, alemtuzumab was administered as a part of conditioning regimen. It is possible that the strong immunosuppressive effect of alemtuzumab suppressed inflammatory condition in the patient, played a role in the favorable outcome in the patient. The use and optimal dosing of alemtuzumab in HCT for LAD-I are the subjects for the future studies.

In the present case, NK cell reconstitution was seen at as early as 1 month post-HCT, and B-cell recovery was observed at 2 months post-HCT. T-cell recovery was lastly observed. B-cell generation was also evident from the increase in KRECs post-HCT. T-cell-memory phenotype skewing and inverted CD4/CD8 ratio persisted until 5 months post-HCT. These posttransplant lymphocyte reconstitutions were similar to previous reports (24–28). Particularly, the rapid expansion of NK cells may be partly caused by comorbid CMV infections, and delayed T-cell reconstitution may have been affected by the relatively high dose of alemtuzumab in our case.

Accumulating evidence suggests that PT-CY is safe and feasible in children. However, most experiences come from HCT for hematologic malignancies. Use of PT-CY in nonmalignant diseases, such as IEI, requires perspectives different from that in malignant diseases. First, since nonmalignant diseases do not require the anti-cancer effect of cyclophosphamide, toxicities of cyclophosphamide need to be considered. The myeloablating effect of PT-CY should be studied to optimize conditioning with PT-CY. The optimal dosing of PT-CY in infants should also be established. Second, the strong immunosuppressive effect of PT-CY needs to be considered. Patients with IEI often have infectious complications. PT-CY is usually accompanied by serotherapy with alemtuzumab or anti-thymocyte globulin to prevent cytokine release syndrome. The concomitant use of serotherapy results in additional immunosuppression. The infectious comorbidities and the effect of strong immunosuppression by PT-CY should be carefully assessed. The long-term toxicities of PT-CY need to be addressed in future studies.

## Data availability statement

The datasets for this article are not publicly available due to concerns regarding participant/patient anonymity. Requests to access the datasets should be directed to the corresponding author.

## Ethics statement

The studies involving human participants were reviewed and approved by the ethics boards of the Tokyo Medical and Dental University. Written informed consent to participate in this study was provided by the participants’ legal guardian/next of kin. Written informed consent was obtained from the minor(s)’ legal guardian/next of kin for the publication of any potentially identifiable images or data included in this article.

## Author contributions

MY wrote the manuscript. DT performed genetic analysis. SE, TK, DN, NM, TI, HF, YM, KO, and HT provided clinical information. TMN, MT, KI, and TM provided critical discussions. HK conceptualized the study and revised the manuscript. All authors contributed to the article and approved the submitted version.

## Funding

This study was supported by MEXT/JSPS KAKENHI (grant number: 22K07887) to HK.

## Acknowledgments

We thank the patient and her parents for participating in this study and Naomi Terada, Maki Yamazaki, Yin Yi, and Keiko Obata for their technical assistance.

## Conflict of interest

The handling editor HM declared a past co-authorship with the author HK.

The authors declare that the research was conducted in the absence of any commercial or financial relationships that could be construed as a potential conflict of interest.

## Publisher’s note

All claims expressed in this article are solely those of the authors and do not necessarily represent those of their affiliated organizations, or those of the publisher, the editors and the reviewers. Any product that may be evaluated in this article, or claim that may be made by its manufacturer, is not guaranteed or endorsed by the publisher.
