# Supplementary material for: Case report: HLA-haploidentical hematopoietic cell transplant with posttransplant cyclophosphamide in a patient with leukocyte adhesion deficiency type I
Source: Front Immunol. 2022 Oct 24;13:1020362. doi: 10.3389/fimmu.2022.1020362 (PMC9638093; doi:10.3389/fimmu.2022.1020362)
Supplement: Supplementary file 1 [file DataSheet_1.docx]

Supplementary Information for

Case Report: HLA-haploidentical hematopoietic cell transplant with post-transplant cyclophosphamide in a patient with leukocyte adhesion deficiency type I

Motoi Yamashita, Shiori Eguchi, Dan Tomomasa, Takahiro Kamiya, Daiki Niizato, Noriko Mitsuiki, Takeshi Isoda, Hanako Funakoshi, Yuki Mizuno, Kentaro Okamoto, Nguyen Minh Tuan, Hidetoshi Takada, Masatoshi Takagi, Kohsuke Imai, Tomohiro Morio, Hirokazu Kanegane

**Correspondence:**Hirokazu Kanegane, MD, PhD
E-mail: [hkanegane.ped@tmd.ac.jp](mailto:hkanegane.ped@tmd.ac.jp)

**This PDF file includes:**

Supplementary Figure 1 and 2

# Supplementary Figure 1. Third episode of omphalitis.

**
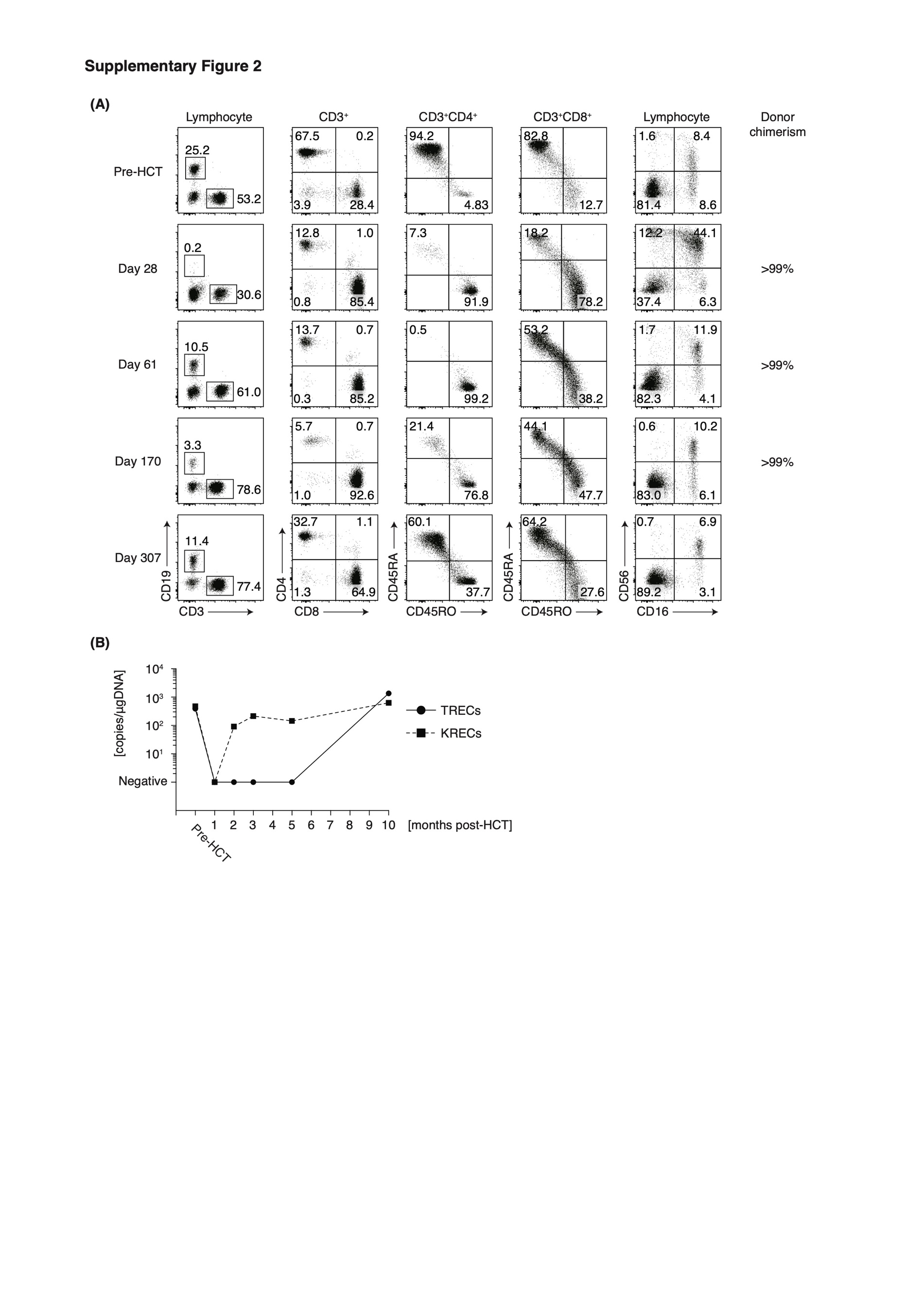
**

**Supplementary Figure 2.** Immune reconstitution after HLA-haploidentical HCT with PT-CY. (**A**) Subset analysis of peripheral blood lymphocytes and donor chimerism pre- and post-HCT. Gating is indicated above each plot. Numbers indicate the percent of cells in each quadrant and indicated populations. (**B**) Peripheral blood T-cell receptor excision circles (TRECs) and κ-deleting excision circles (KRECs) values pre- and post-HCT.
